# Restorative effect of audio and visual elements in urban waterfront spaces

**DOI:** 10.3389/fpsyg.2023.1113134

**Published:** 2023-03-06

**Authors:** Guofeng Zhu, Minmin Yuan, Hui Ma, Zhaoxin Luo, Shegang Shao

**Affiliations:** ^1^School of Architecture, Tianjin University, Tianjin, China; ^2^Research Institute of Highway Ministry of Transport, Beijing, China

**Keywords:** urban waterfront space, restorative environment, visual element, audio element, subjective evaluation, physiological restoration

## Abstract

**Introduction:**

Urban waterfront spaces are often composed of built infrastructures and nature elements. Though citizens could take advantage of these public spaces to relax from daily work, its restorative potential has not been paid enough attention. In this study, the restorative effect and mechanism of different audio and visual elements in urban waterfront spaces was systematically studied.

**Methods:**

At the first stage, restorative potential of waterfront spaces was investigated and different elements with restorative effects were identified through an on-site survey, in which visual and auditory forms of environmental-nature, animal-nature, on-water human activities and on-shore human activities were identified. At the second stage, a series of laboratory experiments were conducted to explore the restorative function of the audio and visual elements.

**Results and discussion:**

It is found that the degree of artificiality of waterfront space was a crucial factor influencing the restoration level of the space, and higher artificiality level of waterfront space resulted in lower level of perceived restoration. However it was available by adding visual and audio elements to the scene to facilitate the restorative effect in waterfront spaces with high-level artificiality. The effects of adding visual and auditory elements on psychophysiological restoration were explored, and elements that should be recommended and restrained were discussed.

**Prospects:**

These findings would provide applicable suggestions for future design and rebuilding of urban waterfront spaces.

## 1. Introduction

With ongoing urbanization worldwide, urban citizens are confronted with increasing environmental pollution and growing stress from daily work and life, which is the vital cause of mental pressure and physical health issues. The concept of “restorative effect,” raised by Kaplan (Kaplan and Kaplan, 1989), offers a new perspective for relieving mental and physical stress. A restorative environment normally contains natural restorative visual and auditory elements that could reduce stress and support recovery from mental fatigue (World Health Organization, 2016), therefore it can facilitate body restoration and enhance people’s wellbeing.

The restorative effect of environments is predominantly covered under two domains of theories, stress recovery theory (SRT) (Ulrich et al., 1991) and attentional restoration theory (ART) (Kaplan, 1995). The SRT indicates that non-threatening natural environments can initiate a restorative process through positive affective responses (Ulrich et al., 1991), while the ART is commonly referenced to identify and restore a cognitive mechanism (Berto, 2005; Kaplan and Berman, 2010). A number of previous literature has reported the restorative effect of visual stimuli from natural visual settings (Nordh et al., 2009; Paddle and Gilliland, 2016; Wang et al., 2016). In addition to this, soundscape was also found to exhibit restorative potential on human’s health (Aletta et al., 2018; Ma and Shu, 2018; Ratcliffe, 2021) and natural sounds were referred by people as restorative factors that supported stress recovery (Cerwén et al., 2016). Moreover, regarding physical restoration, a number of studies suggested that soundscape elements could produce restorative potential on physical features, like skin conductance level (SCL) (Alvarsson et al., 2010) and heart rate (HR) (Hume and Ahtamad, 2013).

In terms of space type, numerical studies exhibited the restorative potential of various environment scenes, such as parks (Nordh et al., 2009), botanical garden (Carrus et al., 2017), playgrounds (Bagot, 2004), university campus (Gulwadi et al., 2019), urban quiet areas (Payne and Bruce, 2019), cemeteries (Lai et al., 2019), Natural environments evidently more often encompassed components that support mental restoration than do man-made environments (Berto, 2005; Berman et al., 2008). However, notwithstanding natural environments could facilitate perceived restoration, natural resources are in some cases not easily accessible to urban citizens. Therefore, exploring the restorative potential from urban built spaces is becoming a new focus. A number of studies suggested that well-designed urban environment could also provide restorative effect on mental pressure reduction (Karmanov and Hamel, 2008; Lindal and Hartig, 2015).

Several studies have reported the restorative potential of water. Presence of water in urban scenes was commonly acknowledged in increasing pleasantness and the sense of intimacy to nature (White et al., 2010). Acoustically, the sounds of river and fountains were proved to be capable of masking urban traffic noise, raising auditory amusement, inducing states of relaxation, and promoting perceived restoration (You et al., 2010; Nilsson, 2013; Leung et al., 2017; Liu et al., 2022). The term “urban blue space” was especially raised referring to water surfaces within a city, and positive effects on physiological health and social-interaction behaviors were suggested in a number of social surveys (White et al., 2010; Völker and Kistemann, 2015; de Bell et al., 2017; Grellier et al., 2017).

Except for the distinction of audiovisual elements from urban green areas, urban parks, cemeteries and other restorative scenes, urban waterfront spaces are unique for the mixture of natural and artificial elements. Natural elements such as water, vegetation and birds often emerge together with man-made elements including urban skyline, built structures and human existence like traffic flow, pedestrian, and activities on shore and water surface, therefore the effect and mechanisms of restoration in waterfront spaces could be different due to the characteristics of their unique audio-visual elements and the degree of artificiality. Besides, in the limited previous studies on restoration of city blue spaces, the relationship between urban waterfront space and health restoration was mainly explored through social surveys, questionnaires and interviews, in which the results could be influenced by various irrelevant factors. On the other hand, existing studies are limited to self-reported evaluations judging the effect of urban waterfront space on health, and the influence on physiological restoration of urban waterfront space is still unclear.

This study focuses on the effects of different audio and visual environmental elements on people’s psychological and physiological restorative effects in urban waterfront spaces with different degrees of artificiality through an on-site survey and a combination of laboratory experiments. To be specific, the following research questions will be explored in the study: (1) Do waterfront spaces with different levels of artificiality produce different restorative effects?(2) What are the effects of adding different types of audio-visual elements in urban waterfront spaces with different degrees of artificiality on the subjective evaluation of environmental restoration and physiological restorative effects, and (3) what are the applicable suggestions on how to improve the restorative effect of urban waterfront spaces with different degrees of artificiality?

## 2. Methodology

**FIGURE 1:**
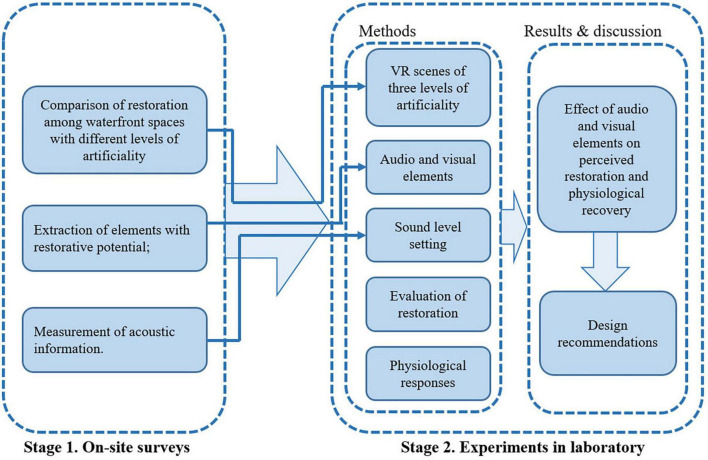
Workflow: Relationship of survey and experiment phases in this study.

In this manuscript, the term “degree of artificiality” was adopted referring to the proportion of human-built elements in the urban waterfront scene. To explore the restorative effects of elements in urban waterfront spaces with different degrees of artificiality, this research was composed of two stages, as illustrated in Figure 1. Stage one was an on-site survey with the actual users in six urban waterfront spaces. The aim the on-site survey was to investigate whether there are differences in the restorative effect of urban waterfront spaces with different degrees of artificiality, and to identify the audio and visual elements in urban waterfront spaces with high restorative potential. Stage two was a combination of laboratory experiments in which subjective evaluation and monitoring of physiological indicators were conducted. During the laboratory experiments the mechanisms of different elements were further explored.

### 2.1. On-site survey

**FIGURE 2:**
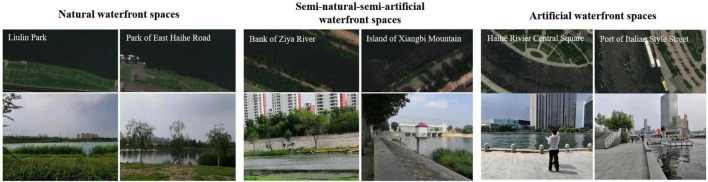
Satellite maps and photos of survey sites.

As a typical city in northern China, Tianjin is distinctive in the representativeness of urbanization and the richness of various waterfront spaces with different degrees of artificiality. Six sites in Tianjin were selected as survey spots. Proportion of vegetation, sky, water surface and artificial pavement and structures in the riverside areas were examined. According to these characteristics, six urban waterfront spaces were classified into three types based on the level of artificial features, namely natural waterfront spaces including Liulin Park and Park of East Haihe Road (in the following abbreviated with NAW), semi-artificial-semi-natural waterfront spaces including Bank of Ziya River and Island of Xiangbi Mountain (SEW), and artificial waterfront spaces Haihe River Central Square and Port of Italian Style Street (ARW). NAW was dominated by vegetation and sky, SEW was mainly composed of artificial paving and built structures, while SEW was a mixture of vegetation and artificial elements, as shown in Figure 2. All the selected sites were not adjacent to main traffic roads, and the dominant sounds were generally from visitors, nature and ships.

A continuous 10 min acoustical measurement was conducted in each site during the questionnaire survey (introduced in the following) using an AWA6228 SPL meter. The acoustical properties of the six waterfront spaces were given in Table 1.

**TABLE 1:** Acoustical properties of six waterfront spaces selected in field survey.

Degree of artificiality	Site	LAeq (dB)	LCeq-LAeq (dB)	LA10 (dB)	LA90 (dB)	LA10-LA90 (dB)
ARW	Haihe River Central Square	54.2	14.7	55.1	51.6	3.5
	Port of Italian Style Street	61.4	9.6	62.4	58.4	4.0
SEW	Bank of Ziya River	57.4	10.5	57.6	50.7	6.9
	Island of Xiangbi Mountain	52.4	11.8	54.7	45.4	9.3
NAW	Liulin Park	51.2	11	56.8	54.4	2.5
	Park of East Haihe Road	49.5	14	50.2	47.2	3.0

The questionnaire was consisted of two questions. The first question asked the respondents to rate the extent of perceived restorative effect of the scene with the following sentence (translated from Chinese):

*Do you think the current environment could make you relax and recover?*

A nine-point bipolar rating scale from completely disagree (1) to completely agree (9) was used for the above question. The second question was semi-open-ended, in which respondents were required to indicate audio and visual elements with restorative potential in each scene from given choices. Meanwhile, respondents were allowed to give supplementary answers. Finally, a total of 300 valid questionnaires were collected from citizens in six urban waterfront spaces.

For the convenience of subsequent laboratory experiment, all elements identified with restorative potential were classified into four categories according to their characteristics, namely environmental-nature category, animal-nature category, on-water human activity category, and on-shore human activity category. Representative elements were selected in each category, and finally six visual elements and seven auditory elements were included in the laboratory experiment to verify their restorative effects, which were boldfaced in Table 2.

**TABLE 2:** Audio and visual elements with high restorative potential in urban waterfront spaces identified in on-site survey.

Category	Visual elements	Auditory elements
Environmental nature	**Small island with plants**, **trees**, grassland	**Sound of stream**, sea waves, sound of wind, **tree rustling**
Animal nature	**Waterfowl**	**Sounds of waterfowl**, **frog croaking**, insect buzzing, birdsong, in the woods
on-water human activity	**Ship**, **boat**, bridge	**Ship whistle**
On-shore human activity	**Fisherman**, **waterside terrace**, people paddling at waterside, people strolling	**Children playing**, **people talking**

Elements included in the laboratory experiment were boldfaced.

### 2.2. Experiment stimuli

For comparison of restorative effects of different audio and visual elements in waterfront spaces with different degrees of artificiality, three scenes of urban waterfront spaces corresponding to types of selected on-site sites in the on-site survey were taken into account, namely NAW, SEW, and ARW as referred before. VR-based environments were proved to be effective in reproducing similar visual and audio experiences as to real scenes in previous studies (Valtchanov et al., 2010; Grellier et al., 2017), therefore scenes of urban waterfront spaces with three different degrees of artificiality were created using Lumion 8.0. To avoid the influence of irrelevant variables, other elements such as sky form and city skyline were kept as consistent as possible among three models.

The original sources for audio experiment signals were selected from the on-site recordings obtained with Head BHS II headset, the BBC radio sound library and the Japanese standard sound library. For each auditory stimuli, the dual-channel sound signal retained the content of each channel recorded on-site, and selected monaural sound sources from libraries were mixed into the two channels with same gains. Adobe Audition 3.0 was used for sound materials mixing, and two principle were taken into account during the mixing process: Firstly, the relative sound pressure level of different sounds were adjusted to ensure that each sound could be heard and recognized clearly and not too loud. Secondly, the frequency and time interval of the occurrences of each sound were adjusted as much as possible to simulate the real environments in order to resemble people’s real feelings. As the average SPL in all waterfront scenes in the on-site survey was 54.4 dBA, the sound level of all the audio stimuli was set at 55 dBA. The stimuli were validated in a pilot experiment to ensure that they could lead to authentic feelings with that in on-site scenes.

Four types of visual elements were added respectively, into the three waterfront scenes in Lumion 8.0. The principle of adding visual elements among three different waterfront scenes was to make the types, numbers, and positions of visual elements as consistent as possible, so that the influence of types, numbers, and positions of visual elements on the experimental results could be minimized to the largest extent. Each panorama was rendered from the same viewpoint, height and perspective, with the size of 8,192 × 4,096 pixels and the resolution of 72 dpi.

**FIGURE 3:**
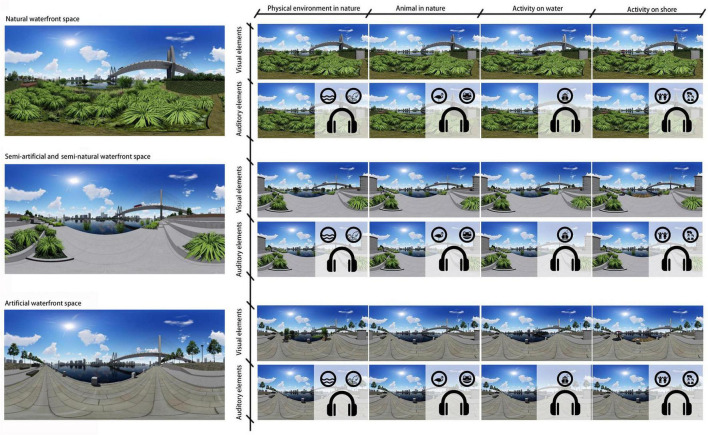
Experimental stimuli with different audio and visual element combinations.

Finally, the four types of audio and visual elements were cross-mixed with each of the three types of urban waterfront spaces, resulting in a matrix of 3 × 8 with 24 experimental stimuli. In addition to control stimuli without any audio or visual elements added, there were 27 experimental stimuli as shown in Figure 3. To avoid excessive duration, the experiments were divided into two groups, corresponding to audio stimuli and visual stimuli, respectively. The procedures of the two experiments were explained in section “Experiment procedure” in detail. During the experiments, the 2D panoramas of three different scenes with various visual elements were presented to subjects through Pico G2 4KS VR headset to create an immersive experience. In the experiments for audio stimuli group, auditory elements were added through AKG K702 dual-channel headphone with a frequency response of 10–39,800 Hz.

### 2.3. Experiment procedure

The changes of electrodermal activity (EDA) and heart rate (HR) were sensitive of external stimuli, and could well reflect the changes of physiological responses of subjects to different experimental stimuli (Valtchanov et al., 2010; Brown et al., 2014; Park and Lee, 2017). Therefore in the laboratory experiments, EDA and HR expressed in micro Siemens (μS) and beats per minute (BPM) respectively, were selected as physiological indicators accounting the physiological restorative effect. EDA and ECG changes were monitored real-time with Biopac MP160 System at 1,000 Hz. HR was derived from the raw ECG recordings using the software package AcqKnowledge 5.0.

In order to control the experiment duration around 1 hour to avoid fatigue and impatience on health indicators, the experiments were divided into two groups, corresponding to all audio stimuli and all visual stimuli, respectively. Each group was consisted of 30 subjects (male 14, female 16 for audio group and male 15, female 15 for visual group). For each group there were 15 stimuli, including three control stimuli with no elements added and 12 stimuli with different audio or visual elements. The stimuli order for each group was randomized.

**FIGURE 4:**
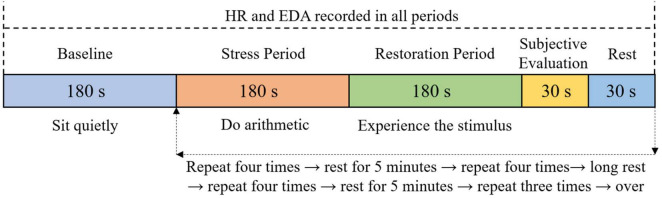
Experiment procedure for each group.

At the beginning of each experiment, the subject was seated in a comfortable chair where the procedure was explained by the researcher. Meanwhile, all the electrodes measuring the physiological responses were attached to the subject’s body to make sure that the gel on each electrode was fully absorbed into the skin. Afterward, a 3-min quiet baseline level was measured. Then a round consisted of four steps began: (1) a 3-min period of oral calculation test (stress period). Specifically, participant were asked to perform continuous subtraction from a number with a step of 7 accurately as soon as possible. The number was randomly selected by researcher from 1,895, 2,020, 3,790, 4,040, 5,685, and 6,060. If he/she did a miscalculation, they would be asked to stop and start their calculation from the beginning. This was assessed to be an effective way to induce stress (Ma and Shu, 2018; Shu and Ma, 2020); (2) a 3-min period of restoration with exposure to one of the experimental stimuli (restoration period), in which subject was asked to sit still and experience the audio or visual stimuli; (3) a 30-sec subjective rating of perceived restorative effect of the current experimental stimulus. While there were existing questionnaire scales regarding restorativeness (Hartig et al., 1996; Herzog et al., 2003; Payne, 2013), the question set in the on-site survey was also proved to be valid in evaluating restorative feelings. Therefore, to avoid long duration and simplify the process, the perceived restorative effect of stimulus was measured with the same question set in the on-site survey; and (4) rest without any stimuli for 30 s. The physiological indicators of stress (EDA and HR) were measured continuously to record their variations during stress period and restoration period. The same procedure was repeated for the other 14 different stimuli. In order to avoid the influence of fatigue on experiment results, each subject was required to have a 10 min rest after proceeded with each four consecutive stimuli. Without counting in rest time length, total time for one experiment was approximately 120 min. The graphic workflow of one typical experiment was shown in Figure 4.

Students enrolled in Tianjin University were recruited as experimental subjects for the experiments. The experiments were performed in a semi-anechoic chamber in Tianjin University. The dimensions of the room was 5.9 × 6.1 × 4.0 m. During the experiment, the background noise level of the room was below 20 dBA.

### 2.4. Statistical analysis

In this study, all the analysis was done within the SPSS software. Single sample Kolmogorov-Smirnov test showed a non-normality of evaluation data, therefore non-parametric methods was applied in all analysis. First of all, the effect of artificiality of waterfront space on restoration from on-site survey was checked using a Kruskal-Wallis one-way ANOVA test. Secondly, there was no influence of baseline since every subject was proceeded with all 15 stimuli tasks in the experiment. Therefore average and standard deviation for each period was calculated for analysis between stress and restoration periods, and the Wilcoxon signed rank test were conducted to examine the differences of restoration. Finally, in the comparison of effects of audio-visual elements in three types of space settings, a meta-analysis was adopted in advance to reduce the effects of baseline of different groups of subjects in the experiment. In all analysis a *p*-value less than 0.05 was used as the criterion to determine significant differences.

## 3. Results

### 3.1. Comparison of perceived restoration in waterfront spaces with different degrees of artificiality

**FIGURE 5:**
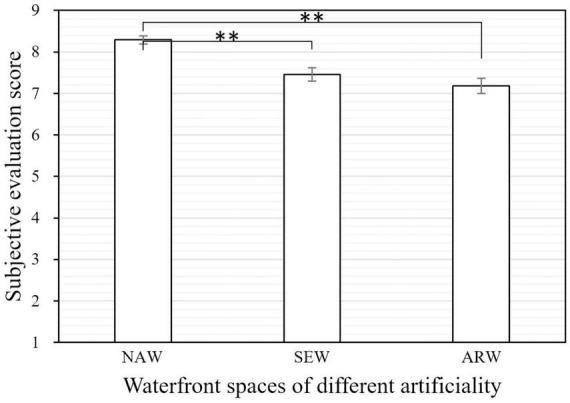
Comparison of restorative evaluation of waterfront spaces with different degrees of artificiality. NAW, natural waterfront space; SEW, semi-artificial-semi-natural waterfront space; ARW, artificial waterfront space. (The error bars represent the standard errors on the averages: ± 1SE). Asterisks indicate statistically significant differences in mean scores (**p < 0.01).

From results of survey questionnaire it was observed that all types of waterfront space exhibited obvious restorative effect on subjective evaluation. By comparison of the difference of restorative effect between natural waterfront space (NAW), semi-artificial-semi-natural waterfront space (SEW) and artificial waterfront space (ARW), there was an attenuation of evaluation on restoration with higher degree of artificiality, with the average value 8.29, 7.46, and 7.18 for NAW, SEW, and ARW, respectively. Kruskal-Wallis one-way ANOVA test showed that the restorative evaluation of NAW was found to be significantly higher than that of ARW (*p* = 0.000) and SEW (*p* = 0.000), while there was no significant difference between the restorative evaluation of SEW and ARW (*p* = 0.984), as shown in Figure 5.

**FIGURE 6:**
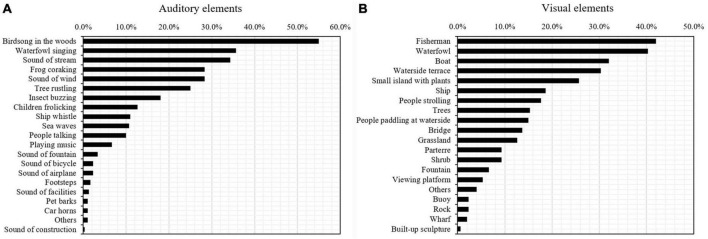
Percentage of auditory elements **(A)** and visual elements **(B)** possessing restorative potential in urban waterfront spaces.

The frequencies of each auditory and visual element considered to have restorative potential existed in urban waterfront spaces were investigated. Twenty auditory elements and 19 visual elements were identified as possessing positively restorative potential, as shown in Figure 6. Among all, 11 visual elements and 11 auditory elements were recognized with relatively high restorative potential (frequencies exceeding 10%) in the urban waterfront space. For the convenience of subsequent laboratory experiments, the audio and visual elements were classified into four categories according to their in-between common characteristics: environmental-nature, animal-nature, on-water human activity and on-shore human activity, as shown in Table 2. One or two audio and visual elements in each category were selected to represent the corresponding category for the following laboratory experiments, which were boldfaced in Table 2.

### 3.2. Effect of audio elements on perceived restoration and physiological recovery

**FIGURE 7:**
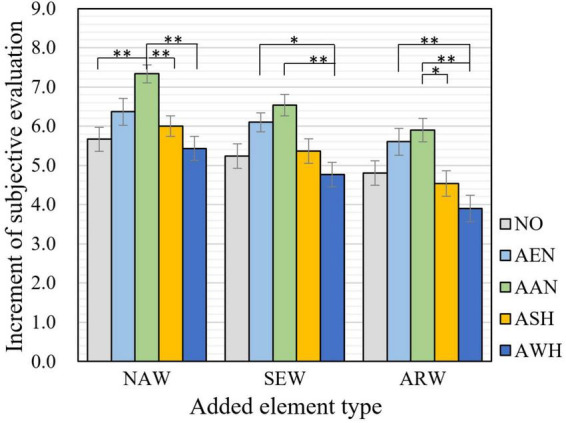
Subjective evaluation of restoration after adding different audio elements in waterfront spaces. NAW, natural waterfront space; SEW, semi-natural-semi-artificial waterfront space; ARW, artificial waterfront space; AEN, auditory elements of environment-nature; AAN, auditory elements of animal-nature; ASH, auditory elements of on-shore human activity; AWH, auditory elements of on-water human activity; NO, no element added. (The error bars represent the standard errors on the averages: ± 1SE). Asterisks indicate statistically significant differences in mean scores (*p < 0.05; **p < 0.01).

The audio elements exhibited different effects on subjectively perceived restoration in three types of waterfront spaces, as shown in Figure 7. As for natural waterfront space (NAW), the addition of auditory elements of animal-nature (abbreviated with AAN) significantly improved the restorative evaluation (*p* = 0.001). Meanwhile, restorative evaluation was significantly higher when adding AAN compared with that of adding auditory elements of on-shore human activity (ASH) (*p* = 0.009) and on-water human activity (AWH) (*p* = 0.000). In semi-artificial-semi-natural waterfront space (SEW), AAN and auditory elements of environment-nature (AEN) were top two effective elements improving the restorative evaluation (*p* = 0.052 and *p* = 0.600, respectively). It is noted that adding of AWH significantly caused lower restorative evaluation compared with adding AEN and AAN (*p* = 0.034 and *p* = 0.001, respectively). As for artificial waterfront space (ARW), though it was not significant, AAN was identified as the most effective element facilitating restoration (*p* = 0.187). The performance of auditory elements was bidirectional, with the addition of AAN and AEN exhibiting a positive effect on restorative evaluation, and a negative effect with the addition of AAS and ASH. Notably, adding of AWH exhibited a significant negative effect compared with adding of AEN (*p* = 0.005) and AAN (*p* = 0.000). It is noted that for most audio elements extracted from on-site survey, the improvement by adding to waterfront space was not significant. This suggests that elements with restorative potential do not necessarily lead to additional restorative effects in laboratory settings.

The effect of different audio elements on physiological recovery, and the difference of HR and EDA data during stress period and restoration period were examined as shown in Table 3. In natural waterfront space, the adding of all auditory elements, including AEN, AAN, ASH, and AWH, resulted in significant recovery effects on HR and EDA. In semi-artificial-semi-natural waterfront space, the adding of AEN, AAN, and AWS led to significant recovery effects on HR and EDA. As for artificial waterfront space the addition of AEN, ASH, and AWH caused significant recovery effects on HR and EDA. In summary, the waterfront stimuli with addition of auditory elements presented pronounced effects on the recovery of physiological response, especially for HR.

**TABLE 3:** Effects on subjects’ HR and EDA after adding different audio elements to NAW, SEW, and ARW.

Space type	Added elements	Period	HR (BPM)		*P*	EDA (μS)		*p*
			**Mean**	**SD**		**Mean**	**SD**	
NAW	AEN	Stress	74.52	12.53	**0.001**	8.72	3.92	**0.001**
		Restoration	72.00	13.04		5.71	3.32	
	AAN	Stress	74.88	12.32	**0.000**	6.90	4.55	**0.003**
		Restoration	70.97	12.48		6.11	4.57	
	ASH	Stress	75.97	13.84	**0.000**	6.23	3.03	**0.035**
		Restoration	72.24	13.20		5.69	3.34	
	AWH	Stress	74.65	14.44	**0.000**	6.18	2.97	**0.003**
		Restoration	71.56	13.14		5.56	2.71	
SEW	AEN	Stress	75.29	13.22	**0.003**	6.57	4.35	**0.019**
		Restoration	72.64	12.92		6.19	4.64	
	AAN	Stress	78.17	19.36	**0.000**	6.52	3.69	**0.002**
		Restoration	73.23	15.64		5.63	3.03	
	ASH	Stress	75.55	13.87	**0.000**	6.23	3.71	0.280
		Restoration	72.62	13.17		5.98	3.72	
	AWH	Stress	75.48	14.05	**0.000**	6.94	4.11	**0.000**
		Restoration	71.89	12.62		6.08	3.59	
ARW	AEN	Stress	75.02	13.31	**0.010**	6.27	2.91	**0.022**
		Restoration	73.02	13.22		5.85	3.07	
	AAN	Stress	75.43	13.25	**0.013**	6.05	3.31	0.074
		Restoration	73.52	14.07		5.61	3.64	
	ASH	Stress	76.17	15.06	**0.004**	6.74	4.38	**0.001**
		Restoration	75.23	14.47		5.81	3.96	
	AWH	Stress	81.44	29.58	**0.001**	6.42	4.23	**0.005**
		Restoration	76.00	21.21		5.73	3.78	

ARW, artificial waterfront space; SEW, semi-artificial-semi-natural waterfront space; NAW, natural waterfront space; AEN, auditory elements of environment-nature; AAN, auditory elements of animal-nature; ASH, auditory elements of on-shore human activity; AWH, auditory elements of on-water human activity. *P*-values indicating significance differences were boldfaced.

### 3.3. Effect of visual elements on perceived restoration and physiological recovery

**FIGURE 8:**
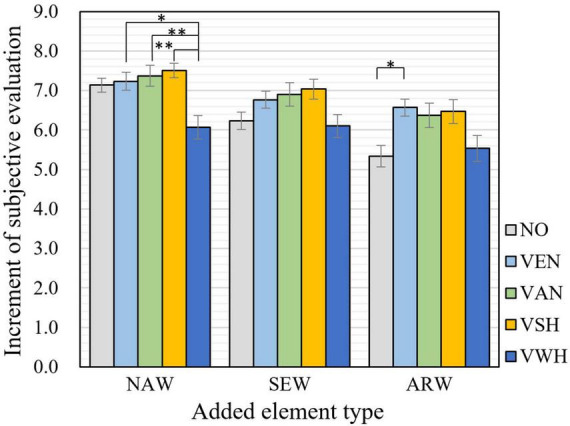
Subjective evaluation of restoration after adding different visual elements in waterfront spaces. NAW, natural waterfront space; SEW, semi-natural-semi-artificial waterfront space; ARW, artificial waterfront space; VEN, visual elements of environment-nature; VAN, visual elements of animal-nature; VSH, visual elements of on-shore human activity; VWH, visual elements of on-water human activity; NO, no element added. (The error bars represent the standard errors on the averages: ± 1SE). Asterisks indicate statistically significant differences in mean scores (*p < 0.05; **p < 0.01).

Comparisons of subjectively perceived restoration with effect of different visual elements in three types of spaces were examined, as shown in Figure 8. In natural waterfront space (NAW), there were insignificant improvements after adding visual elements of environment-nature (abbreviated with VEN), visual elements of animal-nature (VAN) and visual elements of on-shore human activity (VSH). Notably, the adding of visual elements of on-water human activity (VWH) lead to significant lower evaluation scores than that of adding VEN, VAN and VSH (*p* = 0.035, *p* = 0.006, and *p* = 0.004, respectively). For semi-artificial-semi-natural waterfront space (SEW), VEN, NAN and VSH were beneficial in improving restorative evaluation, though no significant effect has been found. In artificial waterfront space (ARW), VEN was identified as inducing a significant effect in facilitating perceived restoration (*p* = 0.045). It is noted that for most visual elements extracted from on-site survey, the improvement by adding to waterfront space was not significant. This suggests that visual elements regarded as restorative by visitors do not necessarily result in significant additional enhancements on restoration in waterfront scenes. Trees and small islands with plants were found to be the more restorative elements compared with waterfowl, ships, boats, fisherman, and water terraces. Meantime, no significant improvements was found in NAW and SEW. This may be due to that the ARW scene lacked natural elements, so the addition of natural visual elements produced a significant enhancement, while there was a high level of naturalization in NAW and SEW, therefore the inducing of natural elements was less effective.

Regarding the effect of elements on physiological recovery, the difference of HR and EDA data during stress period and restoration period was given in Table 4. As for natural waterfront space, the addition of visual elements performed conspicuous effect either on HR or EDA recovery, and no visual elements improved both indices. As for semi-artificial-semi-natural waterfront space, the adding of VSH led to significant recovery effects on HR and EDA. The addition of visual elements had a more pronounced effect on the recovery of HR and a relatively poor effect on the recovery of EDA. Similar results were found in artificial waterfront space, as three types of visual elements exhibited significant effects on HR compared to only one on EDA, which indicated the addition of visual elements had a more significant recovery effect on HR compared with EDA. In summary, scenes added with visual elements exhibited greater restorative effects on HR than on EDA. Only scenes with the addition of views of on-shore human activity produced restorative effect on EDA.

**TABLE 4:** Effects on subjects’ HR and EDA after adding different audio elements to NAW, SEW, and ARW.

Space type	Added elements	Period	HR (BPM)		*P*	EDA (μS)		*p*
			**Mean**	**SD**		**Mean**	**SD**	
NAW	VEN	Stress	76.12	10.72	**0.005**	5.83	2.80	0.113
		Restoration	75.02	9.55		5.35	2.87	
	VAN	Stress	76.75	11.42	**0.001**	5.47	2.99	0.918
		Restoration	74.17	10.46		5.44	2.76	
	VSH	Stress	77.66	10.79	0.086	5.87	2.95	**0.042**
		Restoration	76.13	11.47		5.39	2.91	
	VWH	Stress	78.57	13.74	**0.028**	6.04	3.39	0.688
		Restoration	75.51	12.12		5.76	2.76	
SEW	VEN	Stress	77.12	11.94	0.118	5.82	2.97	0.188
		Restoration	76.44	12.29		5.58	3.10	
	VAN	Stress	78.68	12.24	**0.008**	5.60	2.72	0.175
		Restoration	75.54	11.47		5.33	2.80	
	VSH	Stress	77.94	11.68	**0.001**	5.75	2.82	**0.034**
		Restoration	75.35	12.83		5.29	2.85	
	VWH	Stress	77.30	12.00	**0.005**	6.37	2.84	0.428
		Restoration	76.38	15.37		5.98	2.80	
ARW	VEN	Stress	77.22	13.50	0.057	5.90	3.11	0.820
		Restoration	75.79	12.46		5.79	3.05	
	VAN	Stress	76.98	13.01	**0.000**	6.06	3.13	0.846
		Restoration	73.62	11.67		5.80	3.07	
	VSH	Stress	80.63	16.44	**0.018**	5.97	2.91	**0.002**
		Restoration	75.66	13.01		5.33	2.77	
	VWH	Stress	78.33	12.50	**0.009**	5.67	2.75	0.103
		Restoration	77.39	17.59		5.40	2.85	

ARW, artificial waterfront space; SEW, semi-artificial-semi-natural waterfront space; NAW, natural waterfront space; VEN, visual elements of environment-nature; VAN, visual elements of animal-nature; VSH, visual elements of on-shore human activity; VWH, visual elements of on-water human activity. *P*-values indicating significance differences were boldfaced.

### 3.4. Comparison of restorative effects between audio and visual elements

**FIGURE 9:**
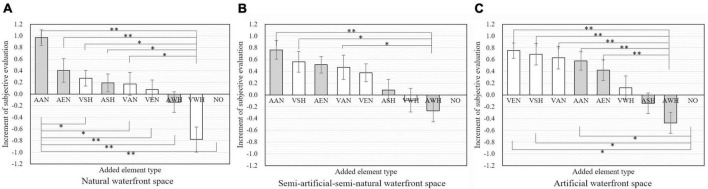
Increment of subjective evaluation of restoration after adding different audio and visual elements in natural waterfront space **(A)**, semi-artificial-semi-natural waterfront space **(B)**, artificial waterfront space **(C)**. AEN, auditory elements of environment-nature, AAN, auditory elements of animal-nature; ASH, auditory elements of on-shore human activity; AWH, auditory elements of on-water human activity; VEN, visual elements of environment-nature; VAN, visual elements of animal-nature; VSH, visual elements of on-shore human activity; VWH, visual elements of on-water human activity; NO, no element added; color green, visual elements; color blue, auditory elements. (The error bars represent the standard errors on the averages: ± 1SE). Asterisks indicate statistically significant differences in mean scores (*p < 0.05; **p < 0.01).

Based on the results of laboratory experiments, the increase in perceived subjective restoration after adding audio and visual elements were compared in Figure 9. It is noted that to reduce the baseline influence in waterfront spaces with different levels of artificiality among two groups of participants, the increment of evaluation score of each experimental stimuli was indicated as the mean value of the difference divided by standard deviation of the evaluation results of corresponding control stimuli in meta-analysis (Glass, 1976; Lin and Bratton, 2015).

Audio and visual elements exhibited different effects in three types of waterfront spaces in subjectively perceived restoration. In natural waterfront space shown in Figure 9A, AAN, AEN, VSH, and ASH were proved to be the most four effective elements enhancing restoration. Besides, restorative evaluation was significantly higher when adding AAN compared with that of adding VAN (*p* = 0.036). These evidences indicated that in natural waterfront spaces auditory representation of biological beings were better than visual forms.

In the semi-artificial-semi-natural waterfront space in Figure 9B, there was no particular distinction on the perceived restoration of the addition of visual and auditory environmental elements. AAN, VSH, AEN, and VAN were top four effective elements improving the restorative evaluation (*p* = 0.107, *p* = 0.150, *p* = 1.000, and *p* = 0.306, respectively). Meanwhile, it is noted that adding of AWH significantly caused lower restorative evaluation compared with adding AAN, VSH, and VAN (*p* = 0.006, *p* = 0.009, and *p* = 0.022, respectively).

As for artificial waterfront space shown in Figure 9C, the addition of visual elements exhibited better effects on perceived restoration compared with auditory elements. VEN, VSH, VAN, and AAN were identified as the most effective elements facilitating restoration (*p* = 0.018, *p* = 0.038, *p* = 0.057, and *p* = 0.035, respectively). Positive effect of all four types of visual elements on perceived restoration was affirmed, while the performance of auditory elements were bidirectional. It is noted that adding of AWH exhibited a significant negative effect compared with adding of VEN (*p* = 0.000), VSH (*p* = 0.001), VAN (*p* = 0.001), AAN (*p* = 0.001), and AEN (*p* = 0.009).

In summary, in natural waterfront space (NAW), the positive effect of adding auditory elements on restorative evaluation was more obvious compared with that in artificial waterfront space (ARW) and semi-artificial-semi-natural waterfront space (SEW); while in ARW, the positive effect of adding visual elements seemed to be more effective. No particular differences between improvement of adding audio and visual elements were found in SEW. The inducing of all audio and visual elements into Figure 9 presented inconsistencies of significance compared with Figures 7, 8. However, it is more straightforward and helpful from the perspective of application, since design practices typically involves combinations of auditory and visual designs rather than only from one sense.

Regarding the effect of different elements on physiological recovery, a similar tendency was illustrated in NAW, SEW, and ARW, as shown in Tables 3, 4. However the adding of auditory elements generally exhibited a better effect on the recovery compared with visual factors. While most of the auditory elements could bring significant restoration of both heart rate HR and EDA, most of the visual elements could only bring significant improvements of recovery effect on HR rather than EDA.

## 4. Discussion

In this study, on-site investigation and laboratory experiments were conducted to explore the restorative effects of different audio and visual elements in urban waterfront spaces with three levels of artificiality on psychophysiological recovery, based on measures of subjectively perceived restoration and physiological indicators.

### 4.1. Restorative effect of urban waterfront space and effective audiovisual elements

Similar to green elements as the principal components in urban green spaces, the restorative potential of water resource in urban waterfront spaces was confirmed in this study. This is consistent with arguments that water resource plays a significant role in the perception of naturalness (Herzog et al., 2000), which has been considered as a crucial factor for facilitation of restorative effect of environments (Herzog et al., 1997; van den Berg et al., 2003; Kahn et al., 2008). In this study, despite the various degree of artificiality among three types of waterfront spaces, all conditions exhibited restorative effect on participants. Meanwhile, natural waterfront space could induce significantly higher perceived restoration evaluation compared with artificial waterfront space and semi-natural-semi-artificial waterfront space, which is coherent with previous researches explaining that natural elements perform better than built environments in mental stress recovery (Morita et al., 2007; Chawla et al., 2014; Hajrasouliha, 2017).

However, although the restorative effect decreased with the increase of artificiality of the waterfront space, in this study it is revealed that restorative potential of highly artificial waterfront spaces could be enhanced by selectively adding visual and auditory elements. Visual elements such as waterfowl, waterside terraces, fisherman, and sounds of water stream, frog croaking, etc. could effectively facilitate restoration from both psychological level and physiological level, enhancing public health of urban environments.

Trees, plants and water surfaces were beneficial in enhancing restorative effect in urban green spaces (Carrus et al., 2017; Wang et al., 2019). In addition to visual elements, in this study it was proved that the soundscape were promising in human psychophysiological restoration, as indicated in previous studies (Medvedev et al., 2015; Aletta et al., 2018; Zhang et al., 2021). Existence of living creatures like waterfowl and crickets were helpful in enhancing restoration in waterfront spaces, therefore, the advantage of animal element in nature was underestimated in similar studies and should be further explored.

Human existences exhibited various effects due to their type and location. Activities on shore such as fishing, children playing were beneficial for restoration enhancement. This is maybe because human at present could bring vitality and liveliness to those spaces (Vogels, 2008; Jo and Jeon, 2020). However, it is noted that the favorable sounds generated by human activities on shore should be within the typical loudness range of urban waterfront spaces, since loud music and noisy play could raise perceived crowd density and cause a decrease in comfort among visitors (Meng and Kang, 2015; Jo and Jeon, 2020). Artificial constructions like water terraces could also facilitate restoration. This is maybe because approaches of touching water could bring more pleasantness than just viewing it (Zhao et al., 2018), since terraces could provide access of getting nearer to water and help people experience water bodily. On the contrary, views and sounds from on-water human activities like ships and boats were proved to be useless or even harmful to waterfront environments. This is likely due to the reason that those activities would disturb the calmness and tranquility of water surface and damage the intactness of landscape (Watts and Pheasant, 2013).

It is also noted that additions of several audio and visual elements were not as effective as found in on-site surveys, for instance water sounds and views of animals. Causes for this could be: (1) the changes of restorative effect caused by audio and visual elements may be depended on the actual experience of the scene and the state of emotion, purpose and expectation of the real users at the moment, and this was not taken into consideration at the experiment stage. (2) Due to the broad view of water in control stimuli, waterfront scene without any added elements had exhibited considerable restorative effect, therefore the effect of adding new elements was limited. This also may be the reason that compared with studies reporting water sound was more effective than other natural sounds (Park et al., 2020), no similar results was revealed in this study. Despite that the pressure increased in this study may not be as stressful compared with work accident videos (Ulrich et al., 1991) or horror videos (Park et al., 2020), it has been proved to be an effective method to cause stress. Therefore the findings were compatible in waterfront spaces for citizens wallowed in heavy daily work.

### 4.2. Design recommendations of audio and visual components

The findings illustrated the restorative effect of a variety of environmental elements in urban waterfront space on subjective evaluations and physiological indicators EDA and HR, respectively. Both audio and visual elements were found to generate positive effects on psychophysiology restoration. In order to induce recommendations for design practice of urban waterfront spaces, the audio and visual elements with positive and negative effects on restoration were further summarized.

Based on the results of experiment, audio or visual elements with top four increment in subjectively perceived restoration were labeled as psychological-positive elements, while elements with least two increment in subjectively perceived restoration were labeled as psychological-negative elements. In the same way, audio or visual elements that could lead to significant recovery in at least one of the physiological indicators EDA and HR were labeled as physiological-positive elements, and elements that produce no significant recovery in EDA or HR were labeled as physiological-negative elements. Finally, elements that are both psychological-positive and physiological-positive were categorized as recommended elements in restoration urban waterfront spaces, as show in Table 5. On the contrary, elements that were either psychological-negative or physiological-negative were categorized as the elements that should be refrained in the design of urban waterfront spaces, as shown in Table 6.

**TABLE 5:** Recommended types of audio and visual elements used in urban waterfront spaces.

	Audio or visual elements	Examples
NAW	AAN	Sounds of waterfowl, frog croaking
	AEN	Sound of stream, tree rustling
	VAN	Waterfowl
	VSH	Fisherman, waterside terrace
SEW	AAN	Sounds of waterfowl, frog croaking
	ASH	Children playing, people talking
	AEN	Sound of stream, tree rustling
	VAN	Waterfowl
ARW	VSH	Fisherman, waterside terrace
	VAN	Waterfowl
	AAN	Sounds of waterfowl, frog croaking

**TABLE 6:** Audio and visual elements that should be refrained in urban waterfront spaces.

	Audio or visual elements	Examples
NAW	AWH	ship whistle
	VWH	Ship, boat
SEW	VEN	Small island with plants, trees
ARW	VWH	Ship, boat
	AWH	ship whistle
	VEN	Small island with plants, trees
	ASH	children playing, people talking
	AWH	ship whistle

As illustrated in Table 5, visual or auditory presentation of animal-nature elements like waterfowl were positive in contributing to restoration of waterfront spaces, regardless of the degree of artificiality. Environmental-nature sounds such as rustling of leaves and water flow turned out to be more effective for restoration enhancing in waterfront spaces with lower degree of artificiality. Interestingly in SEW, the auditory representations of on-shore human activities like children playing and people talking were more beneficial to restoration, compared with visual representations.

From Table 6 it is evidential that ship whistles performed poorly for restoration in all types of urban waterfront spaces, therefore should to be limited. In waterfront spaces of low degree of artificiality, sights of on-water human activities such as boats and ships were also detrimental to restoration. Notably, visual elements of environmental-nature like trees and islands with plants also performed poorly for restoration in SEW and ARW, which indicated that enhancing restorative effect by increasing the greening rate was not always an optimal choice, and different design strategies should be adopted for urban waterfront spaces with various degrees of artificiality.

It is worth noting that in NAW, VWH could cause negative effect on perceived restoration, while in SEW and ARW, existence of AWH showed a significant negative effect compared with results of adding most other audio and visual elements. Therefore, special attention should be paid that elements of on-water human activities should be restricted in urban waterfront scenes.

### 4.3. Implications and limitations

The findings have a number of implications for planning and design in urban waterfront spaces. At the most general level, the findings suggest that waterfront spaces are ideal places to spend leisure time and for recreational activities, restoring people from every day stress and fatigue, which help to enhance physical and mental wellbeing. Moreover, composition of elements is influential to the effect of restoration and therefore should be given explicit attention in planning and design decisions. Specifically, waterfront space with different degree of artificiality should be treated with different design strategies. When the space is originally composed of natural scenery, auditory elements may perform better than visual elements. On the contrary, when the waterfront space is designed in an artificial manner, its restorative potential could be improved from a visual basis. Notably, increasing the degree of naturalness simply by adding natural visual elements to the environment is not effective, and infrastructures providing access to the water such as waterside terraces and fishing implementations may be the better choices.

Subsequently, the advantage of VR technique was exhibited in this study. By constructing a virtual model, VR technique allows for the reproduction of an immersive experience, and moreover allows the researcher to freely alter elements in the scene, thus allowing for targeted research on the effect size brought by variables. Therefore, it owns great promise for application in environmental, physiological and psychological studies.

Potential limitations of the present study are related to the experimental setting and participants. There may be interactive effects between audio and visual elements that influence the restorative effect during perception process, and this should be further explored in future studies. On the other hand, the participants were limited to students aged from 19–28, therefore the demographic factors of subjects were excluded from analysis. Moreover, the age group may be not able to represent the middle-aged and elder urban population. Despite these limitations, the results from this study did provide evidence for elements that facilitate actual restorative effects, from which urban designers could reduce strategies for the design and rebuilding of urban waterfront spaces.

## 5. Conclusion

This study investigated the restorative effect in urban waterfront spaces through on-site survey and laboratory experiments in the following perspectives: (1) whether urban waterfront spaces with different levels of artificiality provide different restorative effects; (2) what are the effects of adding different kinds of audio and visual elements on the psychophysiological restorative effects in urban waterfront spaces with different degrees of artificiality; and (3) audio and visual elements that are to be recommended and to be restricted in order to improve restoration of urban waterfront spaces with different degrees of artificiality. It is found that:

1) Urban waterfront spaces could generate restorative effect regardless of degree of artificiality. The restorative evaluation of natural waterfront space was significantly higher than that of semi-artificial-semi-natural waterfront space and artificial waterfront space, while there was no significant difference between the restorative evaluation of semi-artificial-semi-natural waterfront spaces and artificial waterfront spaces.
2) Restorative effect in natural waterfront space, semi-natural-semi-artificial waterfront space and artificial waterfront space could be enhanced by adding of audio and visual elements. Audio elements of environmental-nature and environmental-animal were positive in increasing perceived restoration in all three types of waterfront spaces, meantime all audio elements exhibited positive effect on physiological indicators including HR and EDA. With regard to visual aspect, elements of environmental-nature, environmental-animal and on-shore human activities were helpful in enhancing perceived restoration in semi-artificial-semi-natural waterfront space and artificial waterfront space, and most of the visual elements could bring significant improvement of restoration on HR. Also it is found that when the artificiality of waterfront space was high, the visual factors had a greater improvement; while when the artificiality of waterfront space was low, the auditory factors exhibited a greater positive effect. In all types of waterfront spaces, the adding of auditory elements had a better effect on the restoration of physiological indicators compared to visual elements.
3) The tables of recommended and restricted elements for restoration were summarized, which could provide a basis for reform and construction of urban waterfront spaces. For instance, in artificial waterfront space, measures such as limiting the sound of boat whistles, establishing fishing platforms and waterside terraces to enrich the activities of people on the shore, breeding waterfowls to raise level of animal nature elements could be taken considerably, rather than simply piling up of trees and plant islands. With regard to natural waterfront space, measures should be considered like limiting of ship whistles, restriction of boats number, as well as inducing sounds of leaves rustling and water flowing, and creating of favorable habitat for animals like waterfowls and frogs.

## Data availability statement

The original contributions presented in this study are included in this article/supplementary material, further inquiries can be directed to the corresponding authors.

## Ethics statement

Ethical review and approval was not required for the study on human participants in accordance with the local legislation and institutional requirements. The patients/participants provided their written informed consent to participate in this study.

## Author contributions

GZ: conceptualization, data curation, formal analysis, investigation, software, visualization, writing – original draft, and writing – review and editing. MY: writing – review and editing, validation, supervision, resources, project administration, methodology, funding acquisition, formal analysis, and conceptualization. HM: writing – original draft, writing – review and editing, validation, supervision, software, resources, project administration, methodology, investigation, funding acquisition, formal analysis, data curation, and conceptualization. ZL: writing – original draft, software, methodology, investigation, formal analysis, data curation, and conceptualization. SS: writing – review and editing, resources, project administration, methodology, investigation, formal analysis, data curation, and conceptualization. All authors contributed to the article and approved the submitted version.
